# Identifying violence against the LGTBI+ community in Catalan universities

**DOI:** 10.1186/s40504-021-00112-y

**Published:** 2021-02-22

**Authors:** Jorge-Manuel Dueñas, Sandra Racionero-Plaza, Patricia Melgar, Paquita Sanvicén-Torné

**Affiliations:** 1 Department of Psychology, Rovira i Virgili University, Tarragona, Spain; 2 Department of Sociology, University of Barcelona, Barcelona, Spain; 3 Department of Pedagogy, University of Girona, Girona, Spain; 4 Department of Geography and Sociology, University of Lleida, Lleida, Spain

**Keywords:** Violence, LGBTI+ students, University students, Sexual orientation, Gender identity, Gender expression

## Abstract

Social struggles have led to the legal recognition of the rights of LGTBI+ people in some countries. Even so, violence against LGTBI+ people is a social problem throughout the world, and has resulted in the vulnerability and victimization of the members of this group. In Spain, no research has been published to date that analyzes this problem in the university context. Considering the scarcity of studies on the identification of this type of violence in Spain, the main objective of this study was to identify violence against LGBTI+ people in Catalan universities. We administered a battery of questions to a sample of 571 university students from six universities in Catalonia (77.8% women) between 17 and 55 years old (*M* = 21.0; *SD* = 3.96). Of the 12 situations of violence presented, psychological violence was identified as the most common type. Within our sample, 61.0% reported either being aware of or having experienced some type of violence related to the university context and motivated by the sexual orientation, gender identity, or gender expression of the victim. The results also show that these types of violence in the university context are rarely reported, especially when they do not include physical violence. This study highlights a previously unreported problem and identifies future research avenues in university contexts.

## Introduction

As the world becomes increasingly progressive, changes have occurred in the rights of sexual minorities, and the last decade has seen a series of victories for LGBT+ communities across the globe (Michelson 2019). But despite all the advancements and acceptance for the LGBTI+ community in some countries today, members of this group remain at high risk of becoming victims of violence for their sexual orientation, gender identity, and gender expression. In fact, in many countries, the human rights of LGBTI+ people are not guaranteed. More specifically, in six countries, sexual minorities are punished with the death penalty, and in 57 others, the maximum sentence for belonging to this community is between 8 years and lifetime imprisonment (Mendos and ILGA World 2019). The LGBTI+ community has been at the receiving end of violence for a very long time in different social spheres (Parker 2017). And violence toward LGBTI+ people can affect them for many years after the aggression occurs (Mawira-Gitari and Walters 2018). It has been reported that LGBTI+ people who are victimized are less likely to complete their studies and, therefore, have fewer job opportunities (Logie et al. 2016).

Furthermore, many gay, lesbian and bisexual people feel the need to hide their sexual orientation to avoid experiences of discrimination in different social settings (Pereira and Costa 2016). Some groups within the overarching classification of LGBTI+ people are more vulnerable than others. For example, trans people subjected to physical and sexual violence have been found to be more likely to attempt suicide, and experienced greater suicidal ideation and increased risk of drug abuse (Testa et al. 2012). In fact, a study conducted with a sample of university students reported that attitudes toward homosexual or bisexual men/women were more positive than attitudes toward transgender people (Copp and Koehler 2017). Additionally, LGBTI+ people belonging to religious minorities or ethnic minorities may be even more vulnerable to violence and discrimination and experience even worse repercussions (Chin et al. 2016; Cyrus 2017; Peumans 2017).

According to reports and studies undertaken in different countries, LGBTI+ students are more likely to be victims of violence and assaults while at university. However, there are no national or autonomous community data that serve to illustrate the current situation of LGBTI+ university students in Spain. These data can be used to establish prevention measures and actions against acts of violence. Therefore, this study is part of the competitive project called Uni4Freedom.Violence due to sexual orientation and gender identity or expression subsidized by Fundació Obra Social la Caixa. It should be noted that this work constitutes the first research project to present data on violence and discrimination in the university environment in Catalonia (Spain).

## Background in educational contexts

The challenges faced by the LGBT+ community in educational institutions has been the focus of much attention in recent years. Several studies have revealed evidence of the discrimination and prejudice that sexual minorities face in educational institutions (Costa et al. 2015; McGinley et al. 2016; Rankin 2005; Coulter and Rankin 2020; Hong et al. 2016). In addition to the family environment, the educational setting is one of the social contexts that most influences psychosocial development and the formation of a child’s identity. More specifically, adolescents develop their identities through social interactions, especially at school. It has been well established that the cultural context of a child’s education is crucial for the development of adolescent identity (Eccles and Roeser 2011). For this reason, educational institutions should be places free of discrimination, aggression, and violence.

As previously stated, situations of violence against sexual minorities are present in most societies in the world, and university settings are no exception. Several studies carried out in different parts of the world show that students belonging to sexual minorities due to sexual orientation, gender identity, or gender expression are more likely to be victimized in different ways throughout the university journey (Costa et al. 2015; McGinley et al. 2016; Rankin 2005), and the odds are even higher for trans students (Coulter and Rankin 2017; Hong et al. 2016; Goodrich 2012). Although the forms of violence to which these people are subjected have been changing and taking on more subtle manifestations, they retain the same intention of causing harm to the LGBT+ person and result in the same consequences for the victim. A more in-depth study carried out by Garvey et al. (2015) looked at the campus climate for LGBT+ undergraduate students at community colleges. Their results revealed perceived inequalities and hostile environments on campus and in the classroom for LGBT+ students, and that the teaching staff was viewed as indifferent to these problems. The authors claim that community colleges have failed to adapt to the growing and changing diversity of their student populations, and suggest that faculty positions on such issues are essential to the student experience, whether positive or negative.

Furthermore, the research conducted by Seelman et al. (2017) with a sample of LGBT+ university students revealed a high prevalence of blatant victimization and microaggressions. These variables were related to low self-esteem and higher levels of perceived stress and anxiety symptoms. In addition, trans students exhibited a stronger negative association between victimization and self-esteem than cisgender students. In the same vein, a study of 8184 Brazilian university students revealed a moderate prevalence of prejudice towards LGBTI+ students; specifically, 2389 reported extreme, high, and moderate levels (Costa et al. 2015).

In the Spanish context, few studies have evaluated the violence, aggression, discrimination, and prejudice experienced by sexual minorities at universities. For this reason, the objective of the present study was to identify students’ perceptions of violence in the Catalan university setting because of sexual orientation, gender identity, or gender expression.

## Method

### Participants

Since the main objective of our research consisted of studying the perception of violence based on sexual orientation, gender identity, or gender expression in university students, data were collected from university students. The sample comprised 571 university students from six public and private universities in the autonomous community of Catalonia (Spain), specifically from the University of Barcelona, University of Girona, University of Lleida, Ramon Llull University, Rovira i Virgili University, and the University of Vic. The age range of the participants was between 17 and 55 years, with a mean age of 21.27 years (SD = 3.95). The participants self-reported their gender identity: 77.8% female, 20.5% male, 0.3% trans person, 0.7% non-binary person, 0.4% person of fluid gender, and 0.4% preferred not to reveal their gender identity. Regarding sexual orientation, the participants identified themselves as follows: 71.9% heterosexual, 17.8% bisexual, 6.3% homosexual, 1.7% pansexual, 1.1% asexual, and 1.2% preferred not to answer the question. The sample size was estimated according to the method proposed by Daniel and Cross (2013) for finite populations in order to obtain a significant sample of the study population. The statistical parameters considered when determining the estimation were (population size = 120,980, margin of error = 5%, confidence level = 97%) = N _adequate_ (469), N _obtained_ (571). The population size corresponds to the total number of undergraduate, master’s, and doctoral students from the six universities under study. Our sample is therefore representative.

To obtain a heterogeneous sample based on the fields of study to which the surveyed student sample belonged, the different disciplines were grouped into three main areas: social sciences and humanities, health sciences, and science and engineering. A proportional distribution was calculated based on the total number of students belonging to each group, and by applying the following criteria: 1) Students from more than one field of study had to be surveyed from each university. 2) Each field of study had to contain students from more than one university.

### Measures

A dichotomous-answer survey was developed for this study. The instrument was designed based on previous findings reported by studies at universities in different parts of the world. The research team conducted a literature review in the Web of Science and SCOPUS databases to select the questions. In addition, the survey was validated by an international expert committee on gender studies and violence against LGBT+ people and by a social affairs committee on LGBTI+ issues made up of members of different associations. This process ensured that the questions were drafted inclusively, reflect situations that LGBT+ people may face in universities, and relate to the objectives of our research.

The questionnaire consisted of four blocks of questions. The first block, A) *sample characteristics*, was designed to collect demographic information. The second block, B) *general identification of violence based on sexual orientation, gender identity, or gender expression*, consisted of dichotomous yes/no questions based on 12 situations for which the person surveyed had to answer whether they considered it violence or not. This block also included dichotomous questions focused on the university environment. In this case, the person had to answer whether they had witnessed any violence within the university context. This dimension consisted of different parameters: physical, psychological, and sexual violence; discriminatory comments; hostile environment, persecution, surveillance, and second-order harassment. The third and fourth blocks were C) *knowledge of the victim’s reaction* and D) *knowledge of measures to prevent violence due to sexual orientation, gender identity, and gender expression in the university context*.

### Procedure

This study was conducted following the principles of the Declaration of Helsinki. It was also approved as under the ethical principles of the University of Lleida and the University of Girona (Catalonia-Spain). An online form was used to administer the survey using the Lime Survey software. The data was encrypted and the computer servers of Rovira i Virgili University (Tarragona-Spain) were used to guarantee the confidentiality and safe custody of the data. Before administering the questionnaire, a pilot test was carried out with undergraduate students to detect errors and ambiguities in the questions. The survey included an introduction, which contained an explanation of the response format for the different questions. It also explained that the data would be completely anonymous and would remain confidential and protected. The participants had to accept the study conditions before participating and express their consent to answer the survey. The effectiveness of surveys administered online has been previously demonstrated. More specifically, the responses to questionnaires on attitudes and perceptions are as valid when administered online as on paper (Mangunkusumo et al. 2006). The online application is useful when inquiring about aspects susceptible to bias based on social desirability and when guaranteeing complete anonymity is imperative. At the end of the survey, information was provided on victim support services at both the university and state levels.

## Results

Table 1 shows the 12 situations of aggression towards the LGBTI+ community and the percentage of participants who considered them a form of violence. All the situations were identified as aggression by more than 86% of the sample. However, situations seven and eight were considered violence by fewer respondents than the other situations. These two situations refer to the concealment of sexual orientation or gender identity for fear of negative consequences. In contrast, insults and teasing, raised in question one, received the highest percentage with 96.35% of the participants regarding them as violence.

**Table 1:** Situations identified as violence in any setting

	Items	% Yes
1	Mocking, insulting, giving homophobic, lesbophobic or transphobic epithets	96.35
2	Exclusion from a specific social activity	89.47
3	Threats, harassment or intimidation	94.10
4	Pressure to keep sexual orientation hidden	93.82
5	Pressure to keep gender expression hidden	92.56
6	Aggressive persecution	92.70
7	Looks of contempt or being stared at with contempt	89.05
8	Avoidance of freely expressing sexual orientation for fear of negative consequences	86.52
9	Avoidance of freely expressing gender identity for fear of negative consequences	86.52
10	Hitting, pushing or exercising physical brutality	93.26
11	Verbalization of homophobic, lesbophobic or transphobic jokes or stereotyped comments about the LGBTIQ+ community	90.73
12	Denial of jobs or work promotions	90.30

The participants were asked if they knew of any violence motivated by the sexual orientation, gender identity, or gender expression of the victim, and 61.2% of the people surveyed stated that they knew at least one case of violence in the university context. The participants were then asked if they themselves had experienced or if they knew anybody who had experienced eight specific situations. Table 2 shows the situations and their corresponding percentages. Having to hide sexual orientation or gender identity obtained the highest percentage of identification (45%), followed by discrimination and humiliating comments (16.83%), and psychological attacks (15.86%). In contrast, the situations with the lowest percentage of identification were leaving university (1.84%) and second order of sexual harassment (1.84%).

**Table 2:** Situations identified as violence in the university context

	Items	% Yes
1	Physical aggression	8.49
2	Psychological aggression	15.86
3	Sexual assault	4.35
4	Avoidance of expressing sexual orientation or gender identity for fear of adverse consequences	45.00
5	Comments, looks, emails, calls, follow-up, waiting outside of class.	8.83
6	Discriminatory, degrading or humiliating comments towards LGTBI people at the university	16.83
7	Second order of sexual harassment	1.99
8	Leaving the university due to a hostile environment	1.84

The third block of the survey was designed to determine whether participants were aware of the victim’s reaction after a case of violence or aggression in the university environment. The answers obtained in this block refer to the most serious of the specific cases that the people surveyed knew about. Therefore, the results do not show all the cases or the students’ average number of known cases. The results revealed that 76.6% of those surveyed recognized the victim’s reaction, while 23.4% stated that they did not know how the victim reacted. Table 3 presents the results and their corresponding percentages. The percentages are not summative because the participants were able to choose different options. Among the respondents, 67.05% who knew of a case of violence in the university context stated that the act was not reported, though it was disclosed to other people. Among the unreported cases, 77.46% maintain that the victim told a friend about the incident. In contrast, 1.16% stated that the victim reported the violence or assault to university staff.

**Table 3:** The reaction of the victim to some type of violence or aggression

	Items	%
1	Cases reported only to the police	5.43
2	Cases reported only to the university	2.33
3	Cases not reported but disclosed to someone	67.05
*Unreported cases*		
3.1	I told my classmates	43.93
3.2	Told a friend	77.46
3.3	Told a family member	21.39
3.4	Told university staff	1.16

## Discussion

The main objective of this study was to identify different types of violence due to sexual orientation, gender identity or gender expression in Catalan universities. Participants were asked to consider 12 situations and state whether each situation constituted violence in the university environment. Our results revealed that all of the situations were identified as aggression by more than 86% of the sample. The situation with the lowest percentage of identification as violence was avoiding expressing sexual orientation and gender identity for fear of negative consequences. In fact, these data coincided with those generated by another question. When participants were asked if they had information about acts of violence against LGBTI+ people, among those who reported being aware an act of violence, 45% reported knowing of a case in which at least one LGBTI+ person hid their sexual orientation or gender identity. This aspect can condition the free expression of one’s gender identity and sexual orientation out of fear of negative consequences. Several studies have found that many LGBTI+ students report being afraid of the negative reactions and homophobia that could occur if they declared their sexual orientation or gender identity (Ellis 2009; Evans and Broido 2002; Lapinski and Sexton 2014; Rankin et al. 2013; Rothmann 2016). This is a problem for LGBTI+ students, because hiding sexual orientation has been linked to mental health problems such as depression and stress (Pachankis et al. 2020).

We also surveyed participants about their awareness of the different types of violence that occur in the university context. The survey revealed that 61.2% of the respondents knew of at least one case of violence in the university context. These data reflect the hostility that university students belonging to sexual minorities may perceive. These results coincide with those from studies conducted in universities in different parts of the world, which report different types of violence directed against LGBTI+ students (Martínez-Guzmán and Íñiguez-Rueda 2017; Ellis 2009; Okanlawon 2020). Although Catalan and Spanish universities have increased their efforts in recent years to protect sexual minorities from discriminatory acts, violence and aggression, apparently these types of attacks have remained in the form of more subtle expressions of violence. These more subtle acts of aggression may go unnoticed (Hong et al. 2016) and may not have physical repercussions, which can make it difficult to eradicate them in the university setting, resulting in harm to the individual and to the social well-being of LGBTI+ students. Indeed, as mentioned above, our data corroborate others’ findings that some students belonging to sexual minorities choose to hide their gender identity and sexual orientation, which can affect their permanence and success at university (Renn 2020), their ability to establish and maintain positive social relationships (Duran and Nicolazzo 2017), and their psychological well-being and mental health (Riggle et al. 2017).

Along the same lines, discriminatory and humiliating comments were the second most identified situation of violence, and psychological aggressions were the third most identified by the respondents. This type of violence can cause the normalization of these types of discriminatory expressions and attitudes in the university community, causing them to be perpetuated over time. This affects the objective well-being, that is, the quality of life of LGBTI+ minorities, as well as their subjective well-being, both cognitive and emotional. Previous research has reported that most of these aggressions and discriminatory attitudes towards LGBTI+ students are perpetrated by students who in turn require accomplices, or silent facilitators, and this combination of actors and situations of violence creates an environment that is hostile, discriminatory and intolerant towards sexual minorities (Clarke 2016; Kheswa 2016; Martin-Storey and August 2016; Rankin 2005; Woodford et al. 2013). To a lesser extent, but no less important, 8.49 of the participants reported being aware of at least one case of physical violence within the university community. The emotional and social consequences of being a victim of this type of violence has been widely studied in the LGBTI+ community, and include emotional anguish, humiliation, fear and depression (Mallory et al. 2017; Davis et al. 2020). This impact is not only experienced by LGBTI+ people who are the victims of physical attacks. People who know of or have witnessed physical attacks may sustain the same psychological repercussions (Gollub et al. 2019). This phenomenon is based on social learning theory (Bandura 1977), which states that people learn by observing behaviors, and that people’s perceptions can be influenced by other people or the consequences that other people’s actions have.

Another aspect that we analyzed was the reaction of the victim to a case of violence or aggression in the university environment. Our data revealed that most cases of violence were not reported to either the university authorities or the police. This can generate a feeling of impunity before the educational community and a feeling of helplessness in the victims (Musalo and Bookey 2014; Konstanski 2011; Vasanthi and Melanie 2017). The scarcity of complaints reflects the lack of visibility and awareness of these events in Catalan universities. Studies conducted with victims of sexual assault show that in the university environment there may be a series of obstacles that make reporting impossible, including, for example, the victim’s fear of the consequences, questioning whether the aggression, discrimination or violence was sufficiently serious to report, not trusting the law or considering that the aggressor(s) powerful enough to delegitimize the complaint, or fear of being blamed for the aggression they have experienced (Holland 2018; Brubaker et al. 2017). Therefore, there is a clear need to promote mechanisms that allow students to lodge complaints without reducing secondary victimization, accompanied by protective services and support for victims. Furthermore, ease of reporting must be accompanied by services that allow victims to seek help.

## Conclusion

The results of the present study reflect different conclusions. First, some situations of violence against LGBTI+ people may go unnoticed or be normalized. For example, avoidance of freely expressing gender identity or sexual orientation for fear of negative consequences were the situations with the lowest percentage of identification of violence. This fact is a problem for the general well-being of LGBTI+ students because having to hide sexual orientation or gender identity can cause discomfort and abandonment of university studies. Future studies should focus on two aspects, 1) design and evaluation of university educational programs that allow the identification of different types of violence, including the most subtle. 2) Analyze and evaluate university policies and good practices on the protection of LGBTI + students. Second, there was a high percentage of violence not reported to the university or the police. This result is worrying because many attacks, discrimination and violence have gone unpunished, this can generate a feeling of helplessness in LGBTI + students, a sense of impunity for the university community, and obvious legal implications. Furthermore, this may be skewing the data on violence, assault and discrimination against LGBTI+ people. Multidisciplinary studies are necessary to analyze these aspects in the university context. Third, this study is the first in Catalonia and Spain to identify violence due to sexual orientation, gender identity and gender expression; the data show expressions of subtle but equally harmful violence.

## Data Availability

Our data is not available for ethical reasons. This study is part of the “Uni4freedom” project, which has been approved by two ethics committees (the University of Lleida and the University of Girona), the ethics committees recommended that the data should not be public and should be kept by the principal researcher of the project in a specific virtual space.

## Abbreviations

LGTBI+: Lesbian, gay, transsexual/transgender, bisexual, intersexual, other minorities due to sexual orientation or gender identity
M: Mean
SD: Standard deviation
